# Reconstructing the evolutionary history of pandemic foot-and-mouth disease viruses: the impact of recombination within the emerging O/ME-SA/Ind-2001 lineage

**DOI:** 10.1038/s41598-018-32693-8

**Published:** 2018-10-02

**Authors:** Katarzyna Bachanek-Bankowska, Antonello Di Nardo, Jemma Wadsworth, Valerie Mioulet, Giulia Pezzoni, Santina Grazioli, Emiliana Brocchi, Sharmila Chapagain Kafle, Ranjani Hettiarachchi, Pradeep Lakpriya Kumarawadu, Ibrahim M. Eldaghayes, Abdunaser S. Dayhum, Deodass Meenowa, Soufien Sghaier, Hafsa Madani, Nabil Abouchoaib, Bui Huy Hoang, Pham Phong Vu, Kinzang Dukpa, Ratna Bahadur Gurung, Sangay Tenzin, Ulrich Wernery, Alongkorn Panthumart, Kingkarn Boonsuya Seeyo, Wilai Linchongsubongkoch, Anthony Relmy, Labib Bakkali-Kassimi, Alexei Scherbakov, Donald P. King, Nick J. Knowles

**Affiliations:** 1 The Pirbright Institute, Pirbright, Woking, Surrey, United Kingdom; 2 Istituto Zooprofilattico Sperimentale della Lombardia e dell’Emilia Romagna, Brescia, Italy; 3 Central Veterinary Laboratory, Department of Livestock Services, Ministry of Agricultural Development, Veterinary Complex Tripureshwor, Kathmandu, Nepal; 4 Department of Animal Production and Health, Gatambe, Peradeniya Sri Lanka; 5 Faculty of Veterinary Medicine, University of Tripoli, Tripoli, Libya; 6 Livestock and Veterinary Division, Animal Health Laboratory, Reduit, Mauritius; 7 Virology department, Institut de la Recherche Vétérinaire de Tunisie, La Rabta, Tunis Tunisia; 8 Virology department, Laboratoire Central Vétérinaire d’Alger, Institut National de la Médecine Vétérinaire, Algiers, Algeria; 9 Laboratoire Regional d’Analyses et de Recherches de Casablanca, Casablanca, Morocco; 10 Regional Animal Health Office No.6, Department of Animal Health, Ministry of Agriculture and Rural Development, Ho Chi Minh City, Vietnam; 11 Department of Livestock, National Centre for Animal Health, Thimphu, Bhutan; 12 Central Veterinary Research Laboratory, Dubai, United Arab Emirates; 13 Regional Reference Laboratory for Foot-and-Mouth Disease in South-East Asia, Department of Livestock Development, Pakchong, Thailand; 14 Laboratoire de santé animale, UMR Virologie, INRA, Ecole Nationale Vétérinaire d’Alfort, ANSES, Université Paris-Est, Maisons-Alfort Cedex, France; 15 Federal Governmental Budgetary Institution “Federal Centre for Animal Health” (FGBI “ARRIAH”), Yur’evets, Vladimir, Russia; 16 Present Address: School of Animal an Veterinary Sciences, The University of Adelaide, Roseworthy, South Australia Australia

## Abstract

Foot-and-mouth disease (FMD) is a highly contagious disease of livestock affecting animal production and trade throughout Asia and Africa. Understanding FMD virus (FMDV) global movements and evolution can help to reconstruct the disease spread between endemic regions and predict the risks of incursion into FMD-free countries. Global expansion of a single FMDV lineage is rare but can result in severe economic consequences. Using extensive sequence data we have reconstructed the global space-time transmission history of the O/ME-SA/Ind-2001 lineage (which normally circulates in the Indian sub-continent) providing evidence of at least 15 independent escapes during 2013–2017 that have led to outbreaks in North Africa, the Middle East, Southeast Asia, the Far East and the FMD-free islands of Mauritius. We demonstrated that sequence heterogeneity of this emerging FMDV lineage is accommodated within two co-evolving divergent sublineages and that recombination by exchange of capsid-coding sequences can impact upon the reconstructed evolutionary histories. Thus, we recommend that only sequences encoding the outer capsid proteins should be used for broad-scale phylogeographical reconstruction. These data emphasise the importance of the Indian subcontinent as a source of FMDV that can spread across large distances and illustrates the impact of FMDV genome recombination on FMDV molecular epidemiology.

## Introduction

Foot-and-mouth disease (FMD) is a highly contagious disease, which affects both wild and domestic cloven-hoofed mammals. It is regarded as one of the most economically important diseases of livestock due to its potential to infect multiple species, affect animal productivity and its ability to rapidly spread within and between geographical regions. The disease is caused by a virus (FMDV; family *Picornaviridae*, genus *Aphthovirus*) which has a non-enveloped virion of icosahedral symmetry encapsulating a positive-sense, single-stranded RNA genome of ~8.4 kb. Similar to most other picornaviruses, the FMDV genome contains a single open reading frame (ORF) flanked by 5′ and 3′ untranslated regions (UTRs). The ORF encodes four structural proteins (VP1 to VP4) and 10 non-structural proteins (L^pro^, 2A, 2B, 2C, 3A, 3B_1_, 3B_2_, 3B_3_, 3C^pro^, and 3D^pol^) being derived from four precursor polypeptides: L, P1, P2 and P3^1^. The synergic effect of fast replication rates, large virus population and lack of proof-reading by the viral encoded RNA-dependent polymerase results in a rapid virus evolution with the ability to generate new genetic lineages^2,3^.

Seven immunologically distinct serotypes of the virus exist: O, A, C, Southern African Territories (SAT) 1, SAT 2, SAT 3 and Asia 1, with serotypes O and A the most widely geographically distributed. Sequence data analyses of one of the virus capsid proteins (VP1) has been successfully used for monitoring virus outbreaks, tracing transboundary movements of virus lineages and to categorise field strains^4–8^. However, the VP1 coding region only comprises ~8% of the FMDV genome and thus by analysing a fragment of the viral genome, a large proportion of the genetic information is not accounted for. With the development of sequencing technologies, obtaining complete or nearly-complete FMDV genome sequences has become more feasible and affordable using both Sanger and Next-Generation sequencing technologies^9,10^. Accordingly, whole genome sequence (WGS) data is being more commonly used for fine scale molecular epidemiology investigations, such as reconstruction of outbreak transmission events^9,11–13^. However, difficulties in obtaining relevant sequence data at the population and geographical levels can affect the resolution reliability of phylodynamic and phylogeographic inferences^14–16^.

The O/ME-SA/Ind-2001 lineage within the Middle East-South Asia (ME-SA) topotype of serotype O was reported in India in 2001^17^. This lineage was subsequently classified into four sublineages named *a*, *b*, *c* and *d*^7^. By 2009, the ‘d’ sublineage of the O/ME-SA/Ind-2001 lineage (Ind-2001d), became the predominant serotype O virus causing epidemics in India^18^ and apparently outcompeting the dominant and long-established O/ME-SA/PanAsia lineage^19^. After a single outbreak was detected in Iran in 2009^7^, the Ind-2001d sublineage was also reported to be the cause of extensive epidemics in North Africa and the Middle East^7,20,21^ in 2013–2014, and in Southeast Asia in 2015^22^, proving its ability to not only become established at the endemic level, but also to rapidly spread over long distances.

Using new and publically available sequences of the VP1 coding region (n = 424) and the whole genome (n = 74) we employed phylodynamics and phylogeographic approaches to trace the global spread of the Ind-2001 lineage, showing the establishment of viral circulations at endemic level outside its initial geographic distribution. We further detailed the evolution and genome structure diversity of the two recently emerging Ind-2001 sublineages (Ind-2001d and Ind-2001e), reporting both evidence of recombination events and of co-circulation of different genetic variants, addressing their impact on molecular epidemiology studies.

## Results

### Transmission History and Phylogeography of the O/ME-SA/Ind2001d and O/ME-SA/Ind2001e FMDV Sublineages

**Figure 1:**
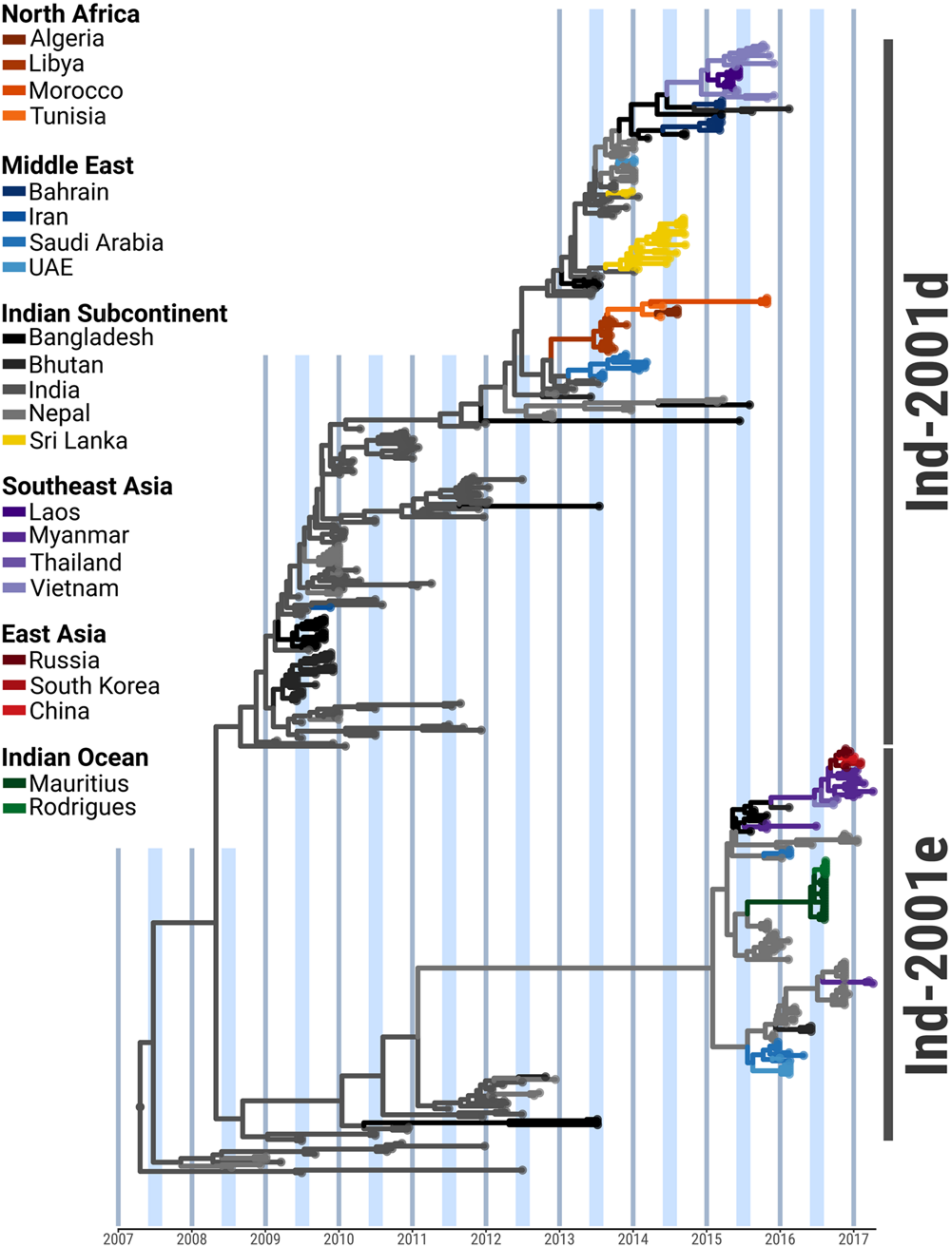
Time-calibrated Bayesian MCC tree inferred for the phylogeographic history of the O/ME-SA/Ind-2001d and O/ME-SA/Ind-2001e FMDV sublineages using n = 424 sequences of the VP1 coding region. Internal branches are coloured according to the most probable country of origin as inferred by the Bayesian discrete phylogeographic method.

Sequences of the VP1 coding region (n = 424), including new (n = 187) and publically available (n = 237) data, of both endemic and epidemic origin were used to reconstruct phylogeographic transitions of the O/ME-SA/Ind-2001d and O/ME-SA/Ind-2001e sublineages across countries (Figs 1, S1; Table 1). The access to viral samples and sequence data for this study was restricted to sample and sequence submissions to the FAO World Reference Laboratory for FMD and from publically available sequence data, which are mainly derived from unstructured and opportunistic sampling. Thus, the inference on precise origins of outbreaks may be affected by sampling biases which impact on the accuracy of phylogeographic reconstructions described in this study. This might be especially the case in the sparsely sampled regions of South Asia and North Africa.

**Table 1:** Median number of reconstructed Markov jumps between geographical regions of origin for the FMDV type O/ME-SA/Ind-2001d sublineage.

	Indian Sub-continent	North Africa	Middle East	Southeast Asia	East Asia	Indian Ocean
Indian Sub-continent	29 (0.97 ± 0.06)	1 (1 ± 0.01)	7 (0.92 ± 0.19)	4 (0.85 ± 0.16)		1 (0.91 ± 0.01)
North Africa		3 (0.99 ± 0.02)				
Middle East			2 (0.95 ± 0.14)			
Southeast Asia				3 (0.97 ± 0.07)	1 (0.64 ± 0.01)	
East Asia					2 (0.98 ± 0.02)	
Indian Ocean						1 (1 ± 0.01)

Posterior probabilities (mean ± standard deviation) are shown in parentheses.

#### Initial endemic circulation within the Indian subcontinent

**Figure 2:**
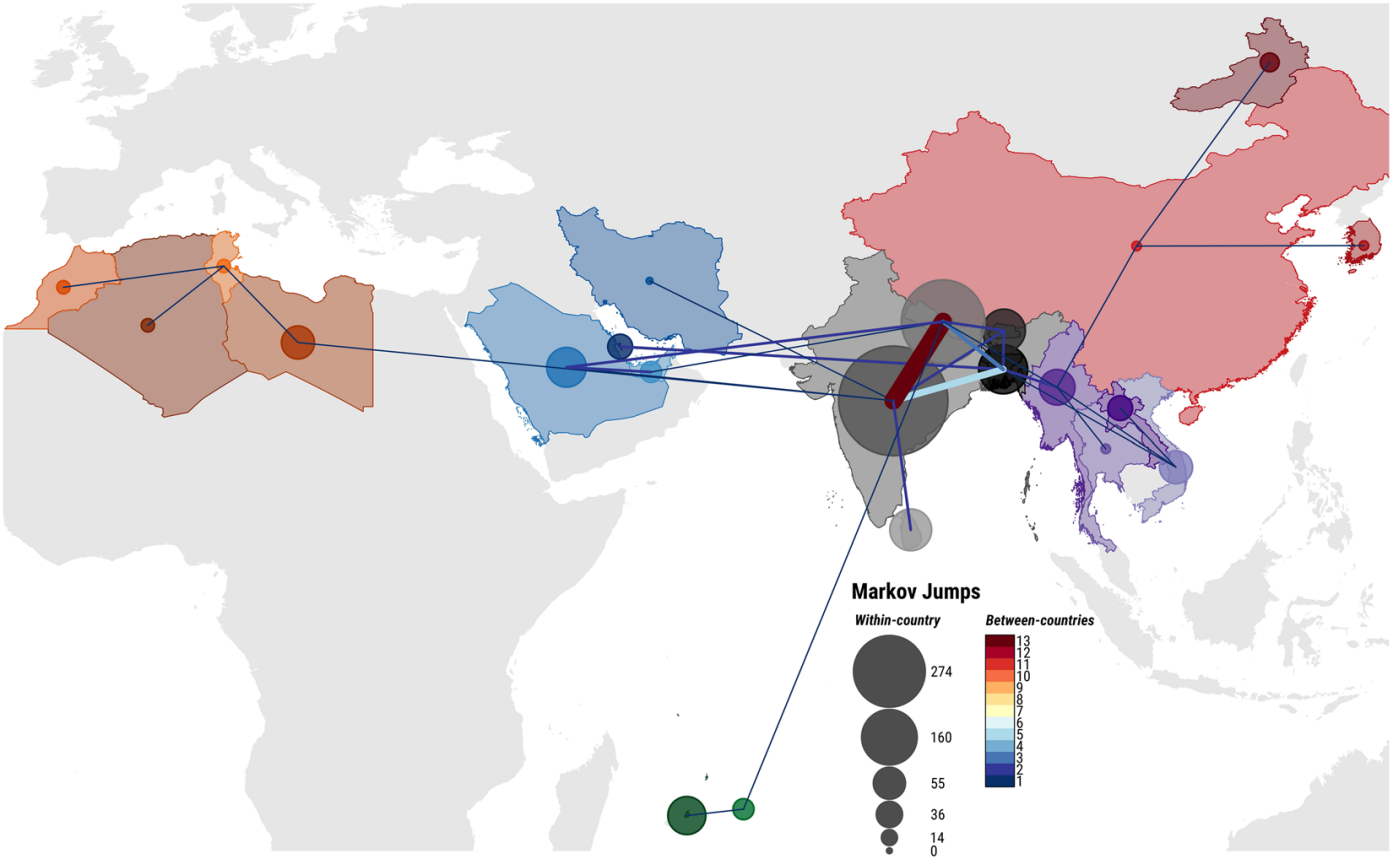
Spatial migration history of the O/ME-SA/Ind-2001d and O/ME-SA/Ind-2001e sublineages across the affected geographical area. Line thickness indicates median number of between-countries transitions, while circled area indicates median number of within-country transitions.

Using a molecular clock, the most recent common ancestor (MRCA) of the O/ME-SA/Ind-2001d and O/ME-SA/Ind-2001e sublineages was predicted to have existed within the Indian subcontinent between 2006 and 2008 [mean April 2007, 95% Bayesian Credible Interval (BCI) March 2006 to April 2008] with high probability [Posterior Probability (PP) = 0.99] (Figs 1, S1). Subsequent evolution resulted in the divergence of the sublineages into two phylogenetic clades. Thus the Ind-2001e sublineage evolved within the Ind-2001d sublineage within the Indian subcontinent. Since 2008, viruses belonging to both sublineages were found to have been regularly co-circulating in India (PP = 1), from where they spread within the Indian subcontinent and, eventually, over longer distances. Until 2012, virus movements were consistently reconstructed between India and Nepal (median number of Markov jumps: 10), Bangladesh (median number of Markov jumps: 3) and less frequently into Bhutan (median number of Markov jumps: 1) (Figs 2, S2 and Table 1). Despite the majority of sequence data available from the Indian subcontinent being classified as O/ME-SA/Ind-2001d, there was evidence of continuous evolution in both sublineages, consistent with an endemic status of these viral sublineages in South Asian countries (Figs 1, S1).

#### Ind-2001d and Ind-2001e virus movements westwards of the Indian subcontinent

The first report of the O/ME-SA/Ind-2001d sublineage outside the Indian subcontinent was a single outbreak in the Kerman Province of Iran in 2009, with inference from the phylogeography analysis ascribing its origin to India (PP = 0.99). Between 2013 and 2015, five independent introductions of the Ind-2001d sublineage have occurred in countries to the west of the Indian subcontinent, followed by two independent introductions of the Ind-2001e sublineage since 2016. In 2013, outbreaks were reported in the Persian Gulf region and affecting Saudi Arabia (MRCA May 2013, 95% BCI April 2013 to July 2013) followed by epidemics in North Africa with outbreaks reported in Libya during the same period (MRCA June 2013, 95% BCI April 2013 to August 2013). Markov jumps analysis supported both the Persian Gulf and North Africa epidemics as originating via two independent introductions of closely related viruses that were circulating in India between 2012 and the beginning of 2013 (PP = 0.99 and PP = 1, respectively) (Figs 1, S1 and Figs 2, S2 and Table 1). Subsequently, the O/ME-SA/Ind-2001d sublineage became established in North Africa, causing a series of related outbreaks in Tunisia (linked with virus movements from Libya, PP = 1) and Algeria in 2014 and Morocco in 2015 (Figs 1, S1). In 2014, a novel introduction was recorded in the Middle East/Persian Gulf region in the United Arab Emirates (UAE) originating in the Indian subcontinent, with the geographic transition likely reconstructed from Nepal (PP = 1). This event was followed by outbreaks reported in Bahrain that were caused by two phylogenetically distant viruses related to contemporary strains circulating in Bangladesh (PP = 1 and PP = 0.70, respectively). In addition, two independent outbreaks due to Ind-2001e viruses were recorded in Saudi Arabia in 2016, one of which spread into the UAE in the same year. Markov jumps inference identified likely transitions of Ind-2001d viruses from Nepal into Saudi Arabia on both occasions (PP = 1).

#### Ind-2001d and Ind-2001e virus movements to the south of the Indian subcontinent mainland

Viruses belonging to both the Ind-2001d and Ind-2001e sublineages were recorded to cause outbreaks in countries south of the mainland Indian subcontinent. The Ind-2001d viruses circulating in India during 2013 (MRCA February 2013, PP = 1) were introduced on two different occasions to Sri Lanka (MRCAs May 2013). In addition, a distinct and unique phylogenetic link within the Ind-2001e sublineage was reconstructed between Nepal and the islands of Rodriguez (MRCA July 2016, PP = 0.99) and Mauritius (MRCA May 2016, PP = 0.90) (Figs 1, S1 and Figs 2, S2 and Table 1).

#### Ind-2001d and Ind-2001e virus movement eastwards of the Indian subcontinent

Sequential apparent Ind-2001d and Ind-2001e virus transitions from Nepal into Bangladesh were, on at least two occasions (MRCAs of October 2013 and May 2015, with PP = 0.98 and PP = 1, respectively), responsible for introductions of Ind-2001 strains into the Southeast Asia region. The first transition of the Ind-2001d viruses, related to strains circulating in Bangladesh (PP = 0.83), was recorded to cause outbreak in Vietnam during 2015 (MRCA November 2014) that spread further into Laos in 2015 (MRCA February 2015, PP = 0.99).

The Ind-2001e viruses were also identified causing outbreaks in Southeast Asia, initially in Myanmar in 2015 (MRCA July 2015) and followed by two further independent introductions to that country in 2017. As revealed by phylogeographic inference, one of the virus introduced during 2017 in Myanmar, which appeared to have originated earlier in time (MRCAs November 2015 and July 2016), spread further to Thailand (PP = 0.69) and Vietnam (PP = 0.75) between June and September 2016, thus becoming established in Southeast Asia (Figs 2, S2 and Table 1). Subsequent outbreaks in East Asia occurred in Mongolia (GenBank accession LC320038; not included in the analyses), South Korea, China and eastern Russia. However, the relatively few sequences available from these regions do not provide enough resolution to resolve the directionality of geographical transitions with high probability (PP < 0.50).

#### Cyclic pattern of the Ind-2001d and Ind-2001e virus occurrence

**Figure 3:**
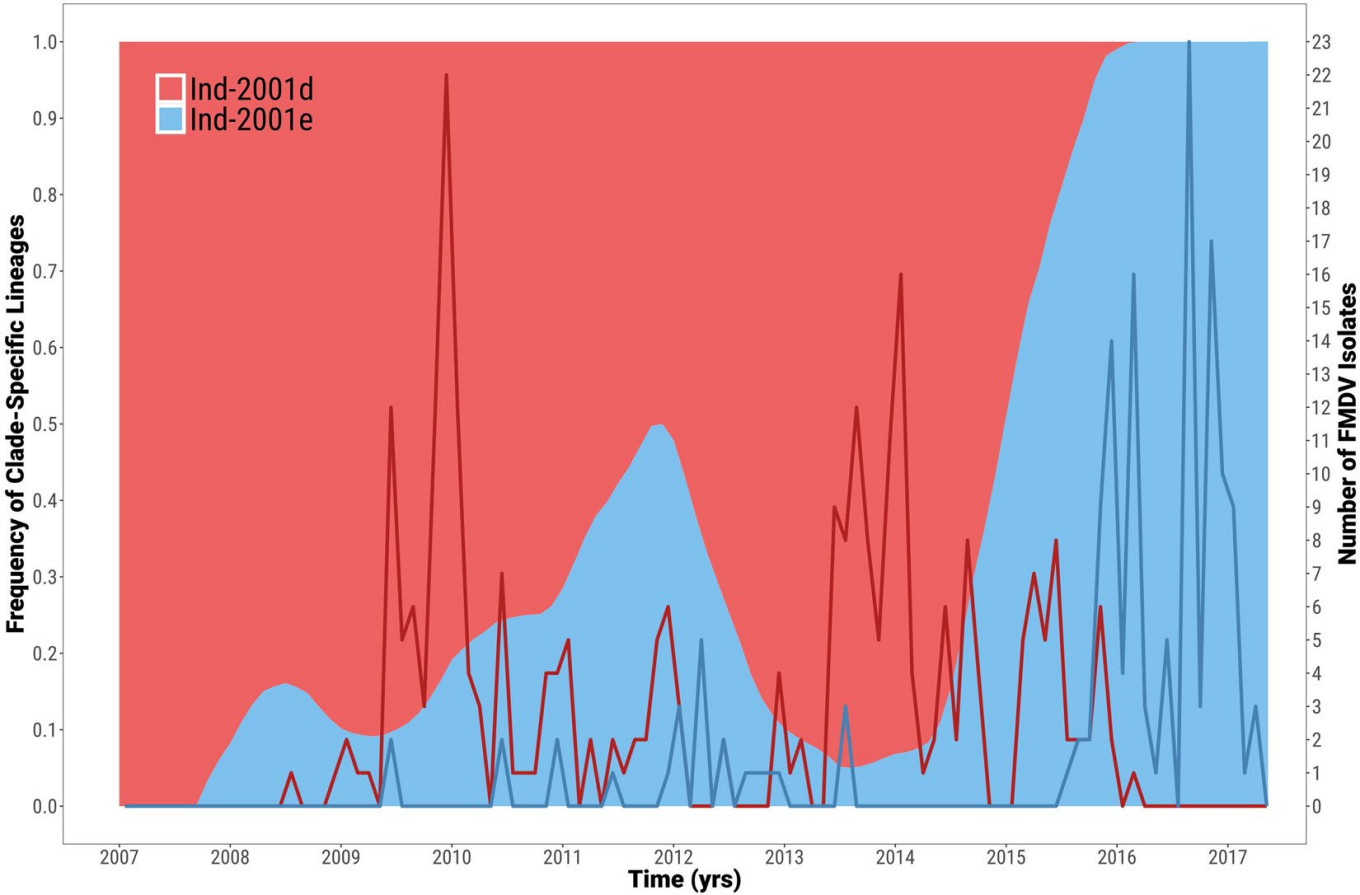
Estimated frequency of O/ME-SA/Ind-2001d and O/ME-SA/Ind-2001e viruses over time. The corresponding monthly temporal trend of isolate sampling from each clade is superimposed by a line graph.

Based on the available sequence data from the VP1-coding region for the Ind-2001d and Ind-2001e samples, the phylogenetic structure provided evidence of cyclical patterns of occurrence of these two sublineages (Fig. 3). In the first phase (between 2007 and 2008), viruses belonging to Ind-2001d were reported more frequently than the Ind-2001e viruses. This trend was found to be reversed in the period between 2009 and 2011. During these two phases, viruses were mainly circulating within the Indian subcontinent. This was followed by a period of increased reporting of the Ind-2001d viruses within the Indian subcontinent (2013–2015) and coincided with the initial notification of the Ind2001d virus outbreaks outside the Indian subcontinent (i.e. the introduction to North Africa, four outbreaks in the Persian Gulf, two outbreaks in Sri Lanka and the later introductions to Southeast Asia). Despite a limited number of Ind-2001e viruses reported during this time, phylogenetic analyses indicated an ongoing evolution occurring within the Indian subcontinent in the two year period between 2013 and 2015. These transmission events, for which sequence data is not available, gave rise to the outbreaks due to the Ind-2001e viruses reported since 2016 (i.e. two independent introduction to the Persian Gulf, the outbreaks in the islands of Mauritius and Rodriguez and three independent introductions into Southeast Asia with the subsequent movement into East Asia). During this time, viruses belonging to the Ind-2001d sublineage continued to be reported in the newly established areas: North Africa and Southeast Asia as well as within the Indian subcontinent. After 2016, viruses belonging to the Ind-2001e sublineage appeared to become dominant (Fig. 3).

### Genome Profile and Evolutionary Features of the O/ME-SA/Ind-2001d and O/ME-SA/Ind-2001e Sublineages

**Figure 4:**
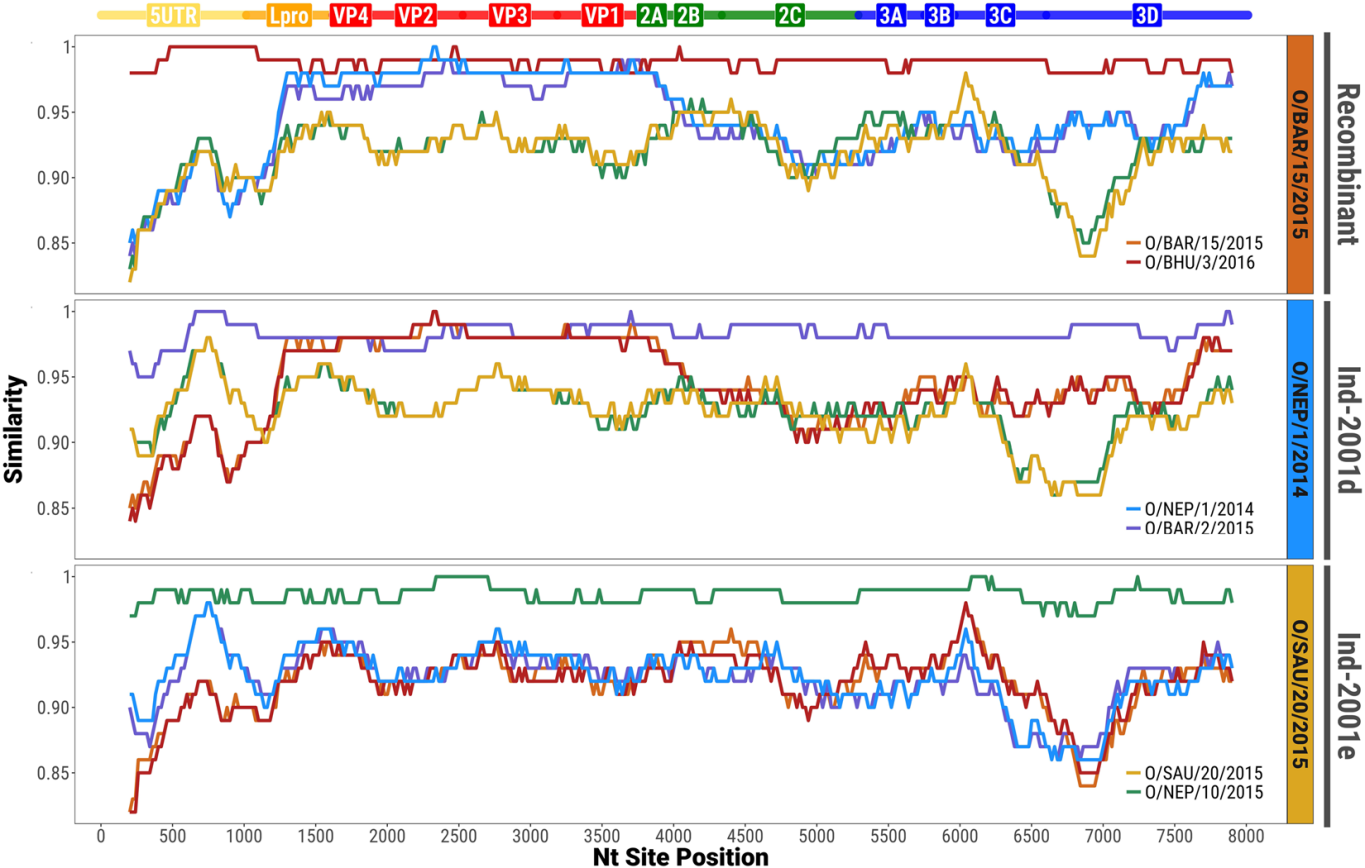
Similarity plots showing recombination within the capsid-coding sequence of the O/BAR/15/2015 and O/BHU/3/2016 viruses. Analyses were performed scanning genome windows of 400 bp of length with a 20 bp step size.

Co-circulation of two sublineages of the O/ME-SA/Ind-2001 lineage was identified based on analyses of the 74 WGSs, including new (n = 59) and publically available (n = 15) data of either endemic or epidemic origin. Two virus sequences (O/BAR/15/2015 and O/BHU/3/2016) differed substantially from the O/ME-SA/Ind-2001d and O/ME-SA/Ind-2001e sublineages in their non-coding (5′ and 3′ UTRs) and non-structural protein-coding (L, P2, and P3) regions. In order to investigate potential recombination, two representative genome sequences were selected from each group (Ind-2001d: O/BAR/2/2015 and O/NEP/1/2014; Ind-2001e: O/SAU/20/2015 and O/NEP/10/2015; the novel clade: O/BAR/15/2015 and O/BHU/3/2016). High similarity (98–100% nt identity) within the respective pairs was observed (Fig. 4). However, in O/BAR/15/2015 and O/BHU/3/2016, the capsid-coding region sequence was found to be most closely related to the Ind-2001d viruses (average nt identity of 97.8 ± 0.01%), whilst the nucleotide sequence of the remainder of the genome was found to be less closely related to both the Ind-2001d and the Ind-2001e viruses (average nt identity of 92.3 ± 0.02%). These findings provided evidence of inter-lineage recombination as a mechanism driving genomic diversity (Fig. 4). A BLAST®^23^ query against all publically available FMDV WGS data did not result in a close genetic match to the novel genome regions of O/BAR/15/2015 and O/BHU/3/2016, making it not possible to identify its parental strain. Even though inter-lineage recombination was not identified for the Ind-2001e viruses (Fig. 4), an increased level of nucleotide sequence variability at the 5′ UTR and 3 C^pro^ and 3D^pol^ coding regions between all groups was evident (average nt identity of 90.8 ± 0.03%).

In addition to nucleotide substitution/recombination variants within the Ind-2001 lineage, different deletions within the non-coding regions were identified. Three different types of deletions of more than 3 nucleotides were observed in the Ind-2001d viruses: (i) a previously reported deletion of 24 nt in the 3′ UTR (O/IND139(302)/2013)^18^, (ii) an 88 nt deletion within the 5′ UTR downstream of the poly(C) tract (O/NEP/18/2013), and (iii) a 72 nt deletion in the s-fragment of the 5′ UTR of viruses isolated during the North African epidemic (O/TUN/1/2014, O/ALG/1/2014, and O/MOR/1/2015). Similarly, deletions in the 5′ UTR were also observed in viruses grouping in the Ind-2001e sublineage: (i) a 74 nt length deletion in viruses causing outbreaks on the islands of Mauritius and Rodrigues (O/MUR/5/2016, O/MUR/9/2016, O/MUR/19/2016, O/MUR/21/2016, and O/MUR/23/2016), and (ii) a 76 nt length deletion in the sequence of the virus isolated in Bangladesh (O/BAN/NA/Ha/156/2013).

### Impact of genome region on molecular epidemiology based on O/ME-SA/Ind-2001d and O/ME-SA/Ind-2001e

**Figure 5:**
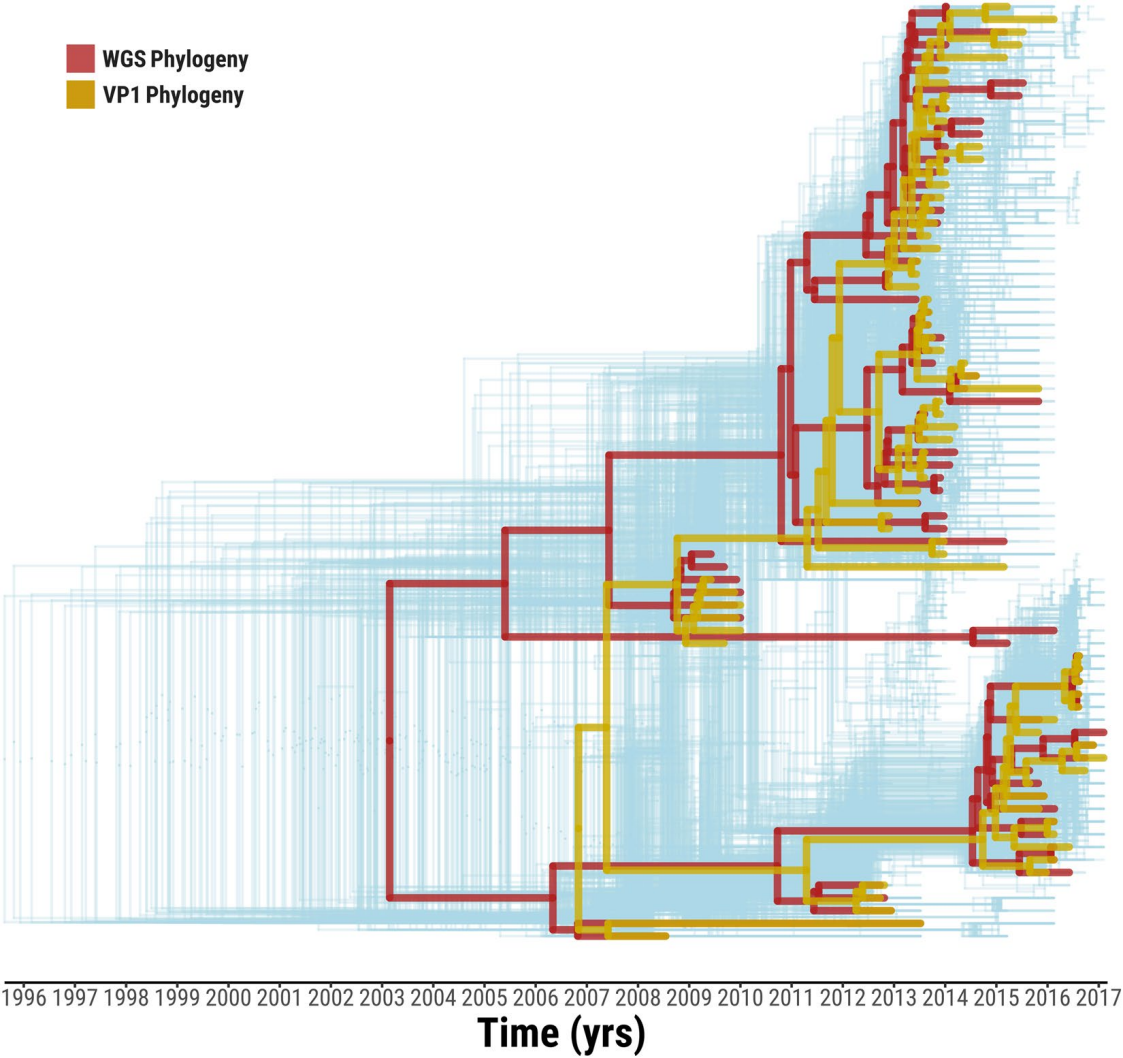
Tree topology variability across the genome of O/ME-SA/Ind-2001d and O/ME-SA/Ind-2001e sublineages. Phylogenetic histories were reconstructed from the 386 trees based on 400 nt genome fragments (step = 20 nt) spanning the whole Ind-2001d and Ind-2001e genome (light blue). The two topological structures representing the phylogenies generated from the WGS (red) and VP1-coding region (orange) sequences are superimposed on the graph.

**Figure 6:**
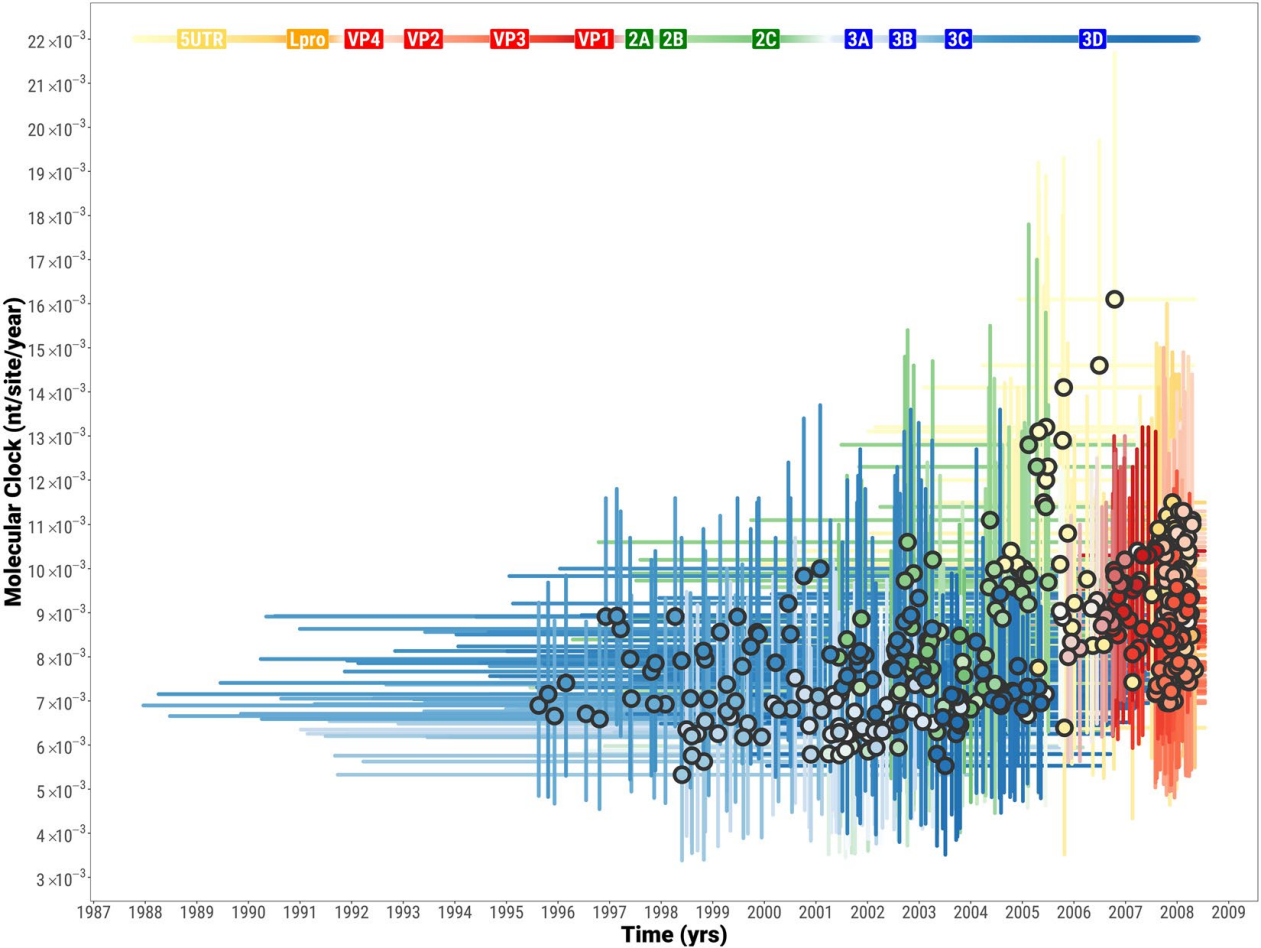
Evolutionary dynamics of each of the O/ME-SA/Ind-2001d and O/ME-SA/Ind-2001e genome fragments. The circles represent the MCRA of each tree as depicted in Fig. 5 with their associated molecular clock. MRCAs and molecular clocks were estimated over the full genomes from 386 genome fragments of 400 nt length at 20 nt steps. 95% BCIs for both the MRCAs and molecular clocks are indicated by line ranges drawn at the x and y axes, respectively.

Time-stamped trees were reconstructed from the 386 alignments of 400 nt-long genome fragments (step = 20) covering the total length of the FMDV genome. These trees were overlaid with phylogenies based on the WGS (n = 74) and the VP1-coding region (extracted from the WGS) (Fig. 5). The WGS and VP1 coding sequences identified two distinct time-stamped phylogenetic topologies. In the phylogeny derived from the VP1-coding region sequences, the two recombinant viruses (O/BAR/15/2015 and O/BHU/3/2016) grouped closely within the Ind-2001d clade. However, in the phylogeny reconstructed using the WGS these viruses were found to form a sister clade sharing coalescence with the Ind-2001d viruses, but distantly related to the Ind-2001e viruses. The resulting ancestry structure of the WGS phylogeny resulted in a shift of the MRCA to an earlier time, estimated at February 2003 (95% BCI February 2001 to January 2005). This did not overlap with the 95% BCI for the MRCA derived from the VP1 data. Multiple possible topologies were revealed from the 386 trees which were in general agreement with the phylogenetic histories derived from the WGS and VP1 sequence data. However, MRCAs estimates varied within a wide time frame (from August 1995 to May 2008) (Fig. 5). MRCA estimates were calculated to be statistically different between genome regions, but comparable within each genome region [F_(13, 372)_ = 158.5, p = 0.000]. The largest difference was observed between trees within the P1 structural and P3 non-structural coding regions (Tukey’s HSD test, average difference of 6.6 years, p = 0.000), and in more detail between VP2/VP3/VP1 and 3C/3D coding regions (Tukey’s HSD test, average difference of 10.1 years, p = 0.000). In contrast to the close clustering of phylogenetic topologies generated from the 400 nt genome fragments representing the structural protein-coding region (P1), the topologies based on genome fragments representing the non-structural protein-coding regions (P2 and P3) were found to be widely dispersed (Fig. 6). This provides further evidence of the impacts of recombination events on the outputs from analyses to reconstruct the evolutionary history of the Ind-2001d and the Ind-2001e sublineages. However, molecular clock estimates were found to be generally similar throughout the genome (values ranging from 5.3 × 10^−3^ to 1.6 × 10^−2^ nt/site/year), with highest values observed within the 5′ UTR/L^pro^ coding regions (average value of 1.0 × 10^−2^ nt/site/year), and lowest values for the 3C/3D coding region (average value of 6.8 × 10^−3^ nt/site/year) (Figs 6, S3). Estimated molecular clocks based on the WGS (n = 74) and the VP1-coding regions (n = 424) sequence data were 5.8 × 10^−3^ nt/site/year (95% BCI 4.6 × 10^−3^ to 6.9 × 10^−3^) and 1.3 × 10^−2^ nt/site/year (95% BCI 1.0 × 10^−2^ to 1.6 × 10^−2^), respectively (Figs 6, S3).

## Discussion

The distribution of FMDV lineages tends to be contained within geographical areas loosely defined as seven regional reservoirs or pools^24^. Pandemics due to single FMDV lineages seldom occur. However, the globalisation in livestock trade and the increased access of developing countries to the global export markets increase the risk that emerging FMDV lineages are likely to be introduced into previously unaffected regions. A previous example was the global spread of the O/ME-SA/PanAsia lineage which emerged from the Indian subcontinent into the Middle East, Southeast Asia, North and South Africa, and into Europe with dramatic economic consequences^17,25^.

In this study, we describe the global spread of two sublineages - the Ind-2001d and Ind-2001e within the O/ME-SA/Ind-2001 lineage of FMDV, and establishment of three new centres of circulation in North Africa, the Gulf States of the Middle East and Southeast Asia. Similar to O/ME-SA/PanAsia, the Ind-2001 lineage emerged from the Indian subcontinent (Bangladesh, Bhutan, India and Nepal). However, virus movement reconstruction within the subcontinent or inference of the geographical source of outbreaks outside of the mainland Indian subcontinent, can be biased by the preferential sampling present in the sequence data analysed due to sparse availability of these data in the public domain. In a number of ways, the spread of this new lineage has parallels with the earlier pandemic distribution of the O/ME-SA/PanAsia FMDV lineage: the emergence from the Indian subcontinent initially westwards into the Middle East/Persian Gulf and North Africa regions, and followed later by an eastward expansion into Southeast Asia and the Far East. The introduction of the virus to FMD-free countries and the long-distance spread (e.g. to the islands of Mauritius and Rodrigues) demonstrates the unpredictable nature of viral epidemics. While many outbreaks of the Ind-2001d and Ind-2001e sublineages were due to single independent introductions of the virus into new regions, some became established within different geographic areas, providing further sources for onward transmission. For example, such events were initially observed in North Africa and more recently in Southeast Asia from where the virus spread further to countries in East Asia. The exact routes of spread is less certain as indicated by lower PP values. However, this extensive spread of the Ind-2001d and Ind-2001e sublineages and their appearance in diverse and distant geographic regions might be linked to increased or altered trading routes, as well as movement of people. Although the data is not yet available, it is also likely that virus-specific factors such as replication and/or transmission fitness across susceptible host species have played an important role in the establishment of this lineage. Similar to the O PanAsia lineage viruses, the Ind-2001 sublineages have also been associated with infection in a wide range of host species, including large (cattle, yak and water buffalo) and small ruminants (sheep and goats), pigs, as well as wildlife (mountain gazelle)^7,22^, a characteristic which might be favourable for the adaption of this lineage to different ecological systems.

Even though deletions within the coding and non-coding regions of the FMDV genome have been previously described^26–28^, the co-circulation of different deletion variants within a single FMDV lineage has not been widely reported. In this study we identified eight different genomic variants within the Ind-2001d and Ind-2001e sublineages among the 74 WGSs analysed, including five different deletions within the non-coding genome regions and recombination involving exchange of the capsid-coding region. However, due to the ad-hoc nature and sampling biases of the sample collection, some of the variants were represented by a single sequence or shared by closely related isolates collected from the same outbreak. However, a deletion within the s-fragment that has arisen in the viruses introduced into North Africa was evident by 2014 and continued to be sustained presumably as an adaptation to the local ecological niche. The recombinant variants (containing the Ind-2001d capsid-coding region within an unidentified FMDV lineage background) was likely a result of reshuffling genome regions from two different parental FMDV lineages co-infecting livestock populations of the Indian subcontinent ecosystem, an evolutionary process that has been previously described^29^. The detection of two closely related recombinant viruses within the Indian subcontinent and in the Middle East indicates that this novel virus may be more widespread than the present sampling suggests.

Variability in tree topology was observed using phylogenies reconstructed from sequential genome fragments extracted from each of the FMDV genome regions. In contrast to phylogenies built using both the non-coding and non-structural protein-coding regions, topologies based on sequences of the capsid proteins were similar to each other. Structural constraints of the FMDV capsid are likely to reduce the virus potential to produce recombination breakpoints within the capsid-coding region. Different genome regions of the Ind-2001d and Ind-2001e sublineages have evolved according to different phylogenetic histories, either influenced by recombination events or other evolutionary processes such as genetic drift. Thus, no single tree topology can accurately capture the true evolutionary relationship of the sampled sequences. In addition, not having one of the parental lineage of recombinant isolates makes the estimation of the MRCA, for the recombinant clade and the two Ind-2001 sublineages, based on WGS data less reliable.

The higher resolution derived from phylogenetic inference based on WGS data has been previously used for reconstructing FMDV transmission on time-constrained and spatially close epidemiological systems^11,13,28^. However, recombination events between lineages, topotypes or serotypes, which may occur in complex epidemiological systems, can impact the ability to ‘phylogenetically trace’ FMD outbreaks^13^. We report that the tree topology resolved for the Ind-2001d and Ind-2001e sublineages using the WGS data, which has been affected by recombination at the genome level of two virus isolates, differs from the structurally constrained topology based on the sequences of the VP1 coding region only. Therefore, the use of the WGS versus the VP1 capsid coding region for molecular epidemiology needs to be carefully considered to minimise these errors

Phylogenetic analyses based on both the VP1 coding region and the WGS data indicate apparent alternating activity of the two genetic clades within the Ind-2001 lineage. Although this observation could be the result of the unstructured and opportunistic sampling of clinical cases from which sequence data were derived. The cyclical activity of FMD viruses has been previously observed at serotype levels (e.g. in Turkey, where outbreaks due to serotype O are often temporally interleaved with outbreaks sustained by viruses of serotype A and Asia 1 origin)^30^ and experimentally (in cell culture) with type O/Asia 1 mixtures^31,32^. While the cyclical occurrence of FMDV serotypes might be driven by herd immunity against individual serotypes and introduction of viruses into naive animal populations, the evolutionary basis underpinning the cyclical occurrence of different genetic clades (and presence of several genomic variants) within a single sublineage is more difficult to explain, although the paucity of publically available sequence data from India from 2014 to date may have influenced these analyses due to sampling biases.

With this study, we aimed at describing the global emergence of the two sublineages of the O/ME-SA/Ind-2001 lineage and resolve its evolutionary history traced across different geographical regions of the world. In addition, we characterised new centres of circulation outside of the Indian subcontinent, in North Africa as well as in the Gulf States and Southeast Asia. The virus was shown to invade geographical areas where multiple FMDV lineages are already present, as well as to FMDV-free areas. The evidence for co-circulation of multiple Ind-2001 genomic variants is provided, including a recombination event involving an unidentified parental strain, presumably derived from different FMDV lineage or serotype endemically co-infecting the livestock population of the Indian subcontinent. Direct comparison of phylogenies based on WGS and VP1-coding sequence data indicates differences in the resolution of these two analytical approaches. As analyses based on WGS data can lead to misrepresentation of the tree topology, and a potential confounding effect for resolving phylogenetic histories, reconstruction of virus movement across and within complex epidemiological systems is recommended to be performed also using structural protein coding sequence data.

## Materials and Methods

### Sequence Data

Sequences of FMDV whole genome (WGS) and of the VP1 coding region were either generated *de novo* from virus isolates held within the repository of the Food and Agriculture Organization of the United Nations (FAO) World Reference Laboratory for FMD (WRLFMD) at the Pirbright Institute, United Kingdom, and FGBI-ARRIAH, Russia, or obtained from GenBank (http://www.ncbi.nlm.nih.gov) (Table S1). The dataset included sequences of the O/ME-SA/Ind-2001 lineage viruses isolated from the Indian subcontinent (India, Nepal, Bhutan, Bangladesh, and Sri Lanka) and collected between 2008 and 2016, as well as from viruses reported isolated between 2009 and 2017 in North Africa (Algeria, Libya, Morocco, and Tunisia), the Middle East/Persian Gulf (Bahrain, Iran, Saudi Arabia, and United Arab Emirates), Southeast Asia (Laos, Myanmar, Thailand, and Vietnam), and East Asia (China, Russia, and South Korea), and the islands of Mauritius and Rodrigues.

The WGS dataset was comprised of a total number of 74 sequences. The new WGS (n = 59) were submitted to GenBank under accession numbers listed in Table S1.

The total number of VP1 coding sequences (n = 424) included new data (n = 187), which were submitted to GenBank under accession numbers listed in Table S1.

### Sequencing

The VP1 coding sequences (n = 187) were determined following the Sanger dideoxy-sequencing methods as previously described^8^.

For whole genome sequencing, viral RNA was extracted from FMDV isolates using the RNeasy® Mini Kit (QIAGEN® Ltd., UK), according to the manufacturer’s protocol. The viral genomes were sequenced using MiSeq technology (Illumina, USA), as previously described^10^. Assembly of raw paired-end reads to consensus-level sequences was undertaken using SeqMan NGen® and SeqMan Pro™ (Lasergene package version 12; DNAStar, Inc., Madison, WI).

The length of the newly generated WGSs ranged from 8045 to 8200 nt and included the full length of open reading frame in all sequences. The mean coverage for all newly generated sequences was 1.4 × 10^3^ and ranged from 1.3 × 10^1^ to 9 × 10^4^. The differences in the length were due to difficulties in resolving the consensus sequence especially at the 5′ UTR and/or the poly(C) regions for some of the samples and/or due to presence of deletions. Accordingly, all WGSs were trimmed for phylogenetic analyses to a length of 8103 bp, excluding 14 nt at the 5′ UTR/poly(C) tract and 78 nt flanking the poly(C) tract.

### Phylogenetic Analysis

Time-resolved phylogenetic trees were estimated using BEAST 1.8.4^33^ employing the general time reversible (GTR)^34^ model of sequence evolution along with gamma-distributed rate variation among sites and 0.5 prior proportion of invariant sites (GTR + G + I). The Bayesian Skyline model was set as tree prior to account for uncertainty in the viral demographic history^35^ and evolutionary rates were allowed to vary across branches using a lognormal uncorrelated relaxed clock model^36^. Choice of the GTR model was based on Bayesian Information Criteria (BIC) results of a statistical selection of the best-fit model of nucleotide substitution using jModelTest 2.1.10^37^. Patterns of FMDV movements across geographical regions were estimated using a discrete-state continuous time Markov chain (CTMC) model, in which transition rates were estimated between each pair of countries assuming an asymmetric non-reversible transition model which employs a Bayesian stochastic search variable selection (BSSVS) procedure^38^. SpreaD3 0.9.7rc^39^ was used to calculate Bayes Factors (BFs) for statistically significant epidemiological links between discrete locations. Markov jumps procedure^40^ was further employed to reconstruct the history of lineage transitions between countries. The Markov Chain Monte Carlo (MCMC) was run for 200 million steps sampling trees every 20000 steps after allowing for a burn-in of 10% of the chain. Estimates had an effective sample size (ESS) of 250 at the minimum and most had ESS greater than 500. Phylogenetic trees were plotted using the *ggtree* package^41^ for R [version 3.4.2; R Foundation for Statistical Computing, Vienna, Austria. (https://www.R-project.org)].

### Genome Profile and Recombination Analyses

A recombination detection analysis was carried out to identify signals of recombination and putative recombinant sequences using SimPlot 3.5.1^42^, setting a sliding window 400 bp wide with a step size of 20 bp. Results obtained were then confirmed by bootscan analysis of 1000 bootstrapped trees generated using the Kimura 2-parameter model^43^. In addition, to further identify variabilities in the topology of phylogenies derived from different regions of the FMDV genome, the n = 74 WGS alignment was trimmed to fragments of 400 bp length obtained at intervals of 20 bp. Each of the resultant n = 386 alignments were then classified into the FMDV genome region of derivation. Time-resolved trees from each alignment were finally reconstructed using BEAST 1.8.4, setting the runs with the same parameters as previously described but excluding the phylogeography inference from the MCMC computation. Statistical analyses were performed in R [version 3.4.2; R Foundation for Statistical Computing, Vienna, Austria. (https://www.R-project.org)], whilst graph were plotted using the *ggplot2* package for R^44^.

## Electronic supplementary material

Supplementary Table 1

Supplementary Figures S1-S3
