# Supplementary material for: Reconstructing the evolutionary history of pandemic foot-and-mouth disease viruses: the impact of recombination within the emerging O/ME-SA/Ind-2001 lineage
Source: Sci Rep. 2018 Oct 2;8:14693. doi: 10.1038/s41598-018-32693-8 (PMC6168464; doi:10.1038/s41598-018-32693-8)
Supplement: Supplementary file 2 — Supplementary Figures S1-S3 [file 41598_2018_32693_MOESM2_ESM.pdf]

Reconstructing the evolutionary history of pandemic foot-and-mouth disease viruses: the impact of recombination within the emerging O/ME-SA/Ind-2001 lineage

Katarzyna Bachanek-Bankowska,[1,*,+] Antonello Di Nardo,[1,+] Jemma Wadsworth,[1] Valerie Mioulet,[1] Giulia Pezzoni,[2] Santina Grazioli,[2] Emiliana Brocchi,[2] Sharmila Chapagain Kafle,[3] Ranjani Hettiarachchi,[4] Pradeep Lakpriya Kumarawadu,[4] Ibrahim M. Eldaghayes,[5] Abdunaser S. Dayhum,[5] Deodass Meenowa,[6] Soufien Sghaier,[7] Hafsa Madani,[8] Nabil Abouchoaib,[9] Bui Huy Hoang,[10] Pham Phong Vu,[10] Kinzang Dukpa,[11] Ratna Bahadur Gurung,[11] Sangay Tenzin,[11,^] Ulrich Wernery,[12] Alongkorn Panthumart,[13] Kingkarn Boonsuya Seeyo,[13] Wilai Linchongsubongkoch,[13] Anthony Relmy,[14] Labib Bakkali Kassimi,[14] Alexei Scherbakov,[15] Donald P. King,[1] and Nick J. Knowles,[1]

Supplementary figures S1-S3

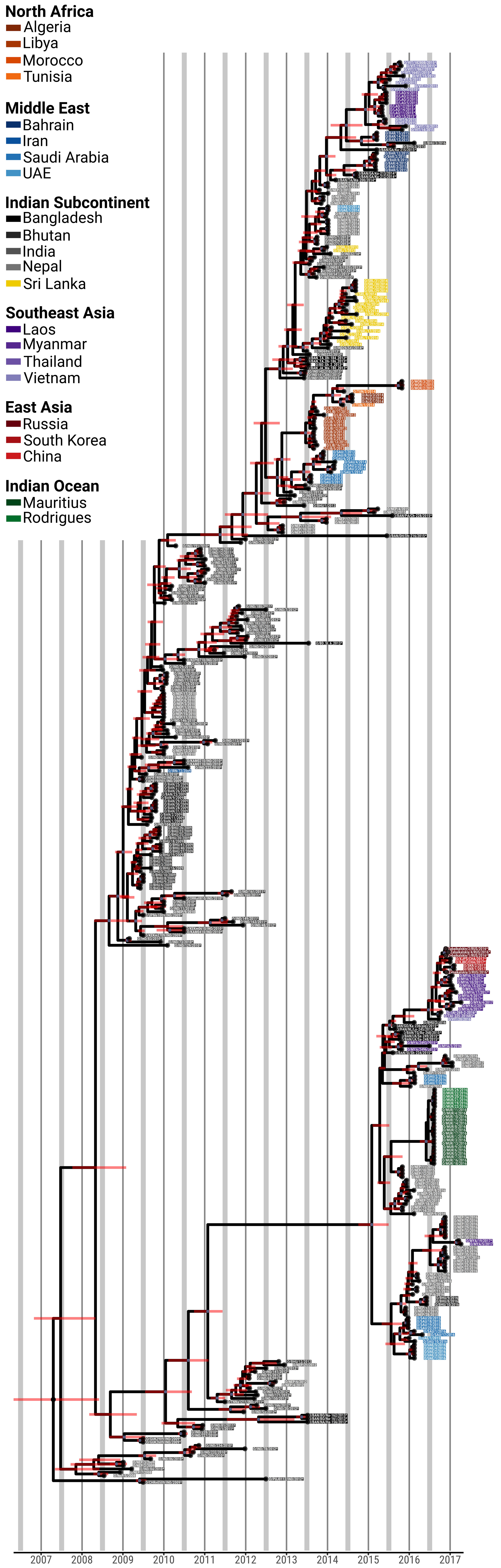

Figure S1 - Time-calibrated Bayesian MCC tree inferred for the O/ME-SA/Ind2001d and O/ME-SA/Ind-2001e FMDV sublineages using n=424 sequences of the VP1 coding region. Nodes with a posterior support of >0.75 are represented in blue. Node bars define the uncertainty in date for each node (95% BCI). The WRLFMD isolate name used for labelling the tips is coded as follows: international country code/the consecutive number of the isolate from a given country in a given year/the year of collection.

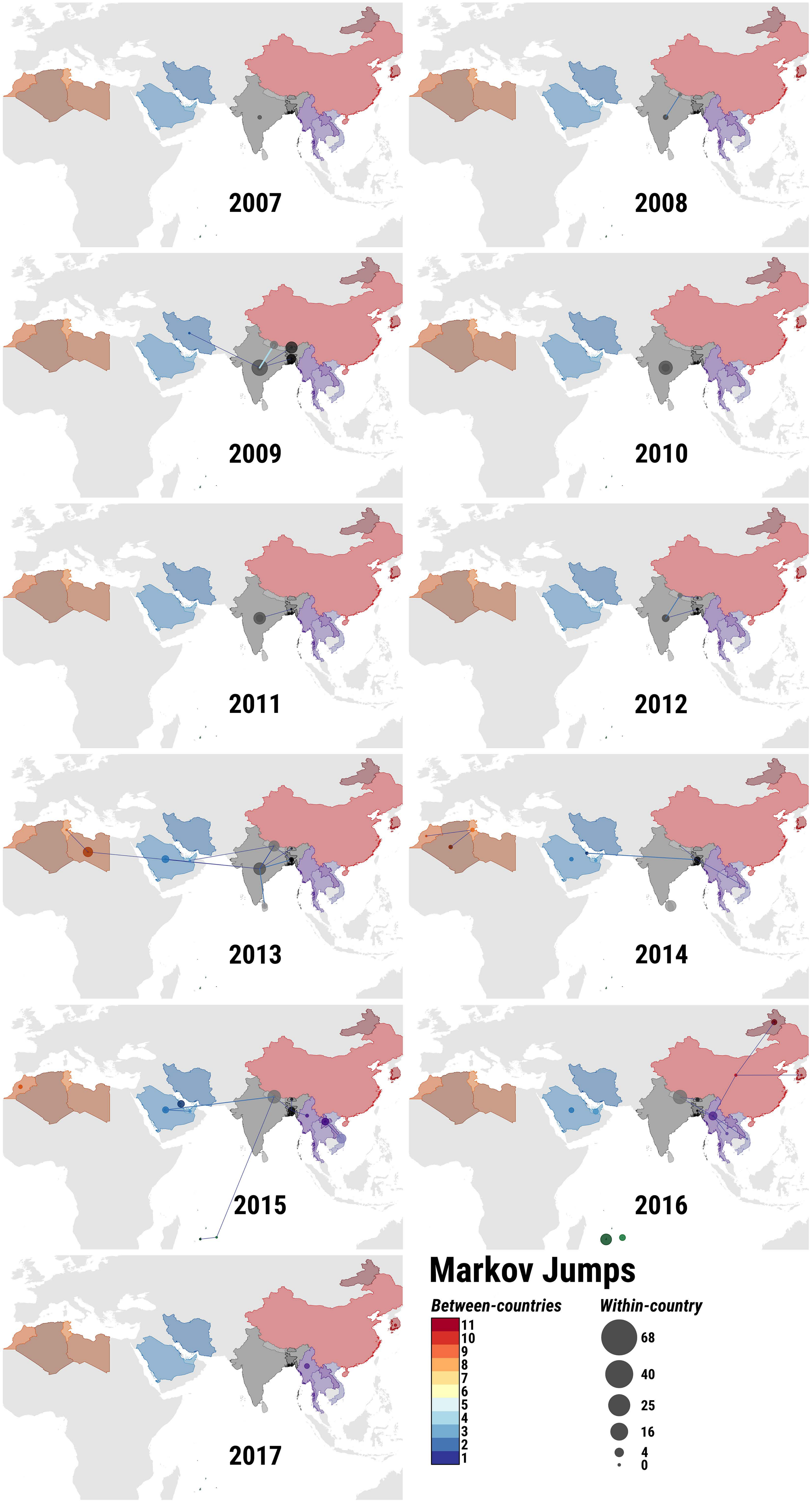

Figure S2 - Spatial migration history of the O/ME-SA/Ind-2001d and O/ME-SA/Ind-2001e sublineages spread across the affected geographical area by year of reporting. Line thickness indicates median number of between-countries transitions, whilst circle area indicates median number of within-country transitions.

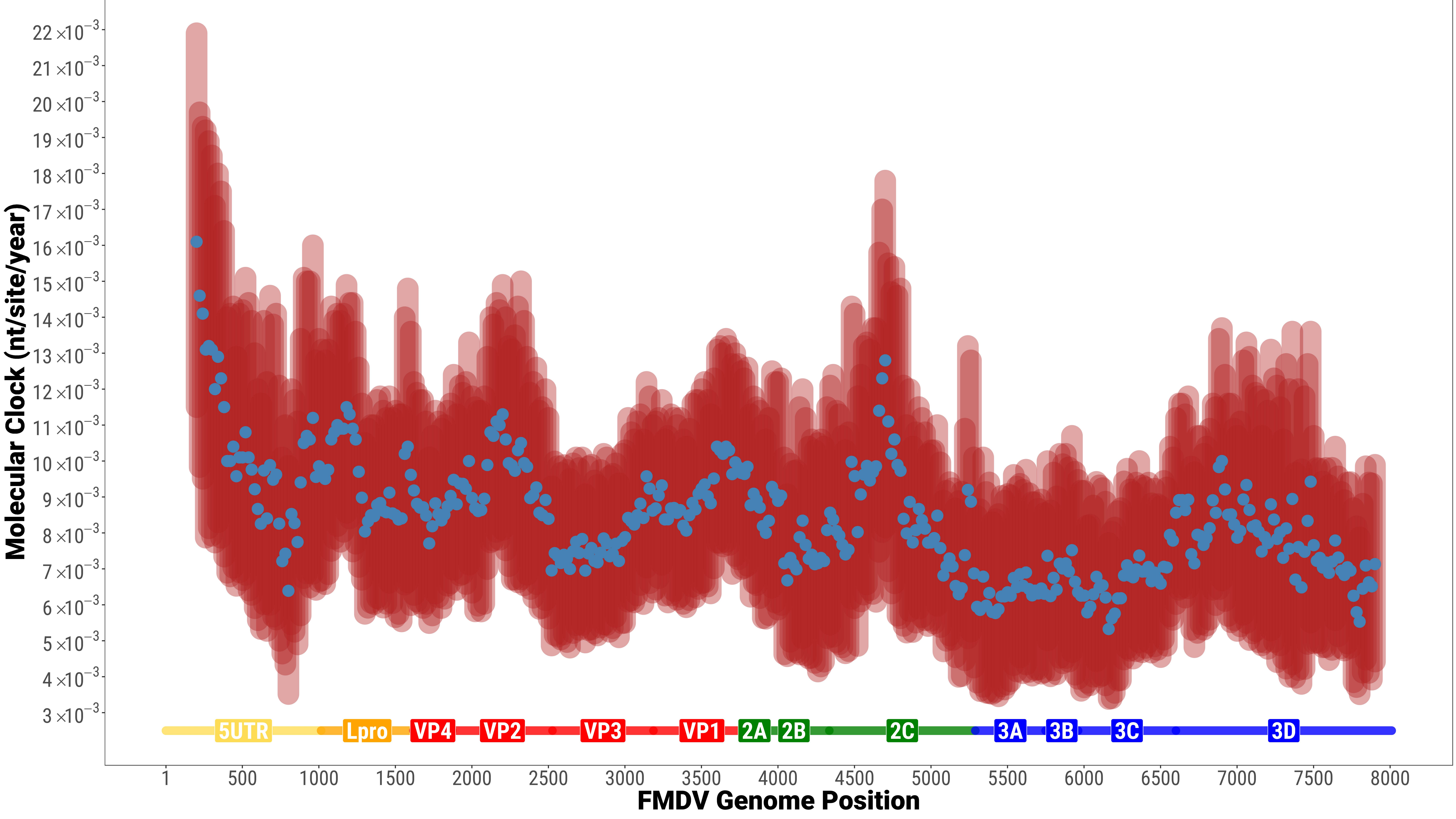

Figure S3 - Estimates of the molecular clock across the O/ME-SA/Ind-2001d and O/ME-SA/Ind-2001e genome. Analyses were based on genome segments of 400 bp of length extracted at 20 bp steps. Mean values are represented by blue points, whilst the 95% BCIs are reported as red segments.
